# Stomach cancer elective surgery morbidity and mortality at 90-Day (Hold Study): a prospective, international collaborative cohort study

**DOI:** 10.1007/s00423-025-03890-7

**Published:** 2025-11-14

**Authors:** Claudia Neves-Marques, Mohamed Abulazayem, Geoffrey Yuet Mun Wong, Ricardo David Maldonado, Yirupaiahgari Viswanath, Alex Boddy, Claire Donohoe, Juan Pablo Scarano, Alessandro Martinino, Amaar Aamery, Nuriddin Abdulkhakimov, Essam Eldien Abuobaida, Ulaş Aday, Khayry Al-Shami, Faris Alhajami, Ana Almeida, Mohammad Badr Almoshantaf, Hassan Ahmed, Martín Andrada, Abdul Wahid Anwer, Ahmed K. Awad, Efstratia Baili, Oussama Baraket, José Barbosa, Cara Baker, Ashraf Bakri, Zdenko Boras, André Caiado, Can Cayirci, Giacomo Calini, Pasquale Cianci, Esin Cinal, Christos Chouliaras, Elif Colak, Maria Teresa Correia, Beatriz Costeira, Viktoria Davletshina, Fabrizio D’Acapito, Turgut Donmez, Evgeniy Drozdov, Giorgio Ercolani, Sarnai Erdene, Ergin Erginöz, Samantha Rocha Ferreira, Marta Fragoso, Massimo Framarini, Aysuna Galandarova, Laurent Genser, Ilya Gorohov, Jan Grosek, Silvia Guerrero, Ismail Hasırcı, Arturan Ibrahimli, Javier Ithurralde-Argerich, Mehmet Karabulut, Takahiro Kinoshita, Ibtissam Bin Khalid, Khurram Khan, Shahid Khattak, Vladimir Khomyakov, Wiktor Krawczyk, Almu’atasim Khamees, Alexander Kostrygin, Jurij Košir, Zbigniew Lorenc, Jorge Milhomem, Gadi Marom, Ana Melo, Abdelkader Menasria, Serhat Meric, Francesk Mulita, Andrea Muratore, Taryel Omarov, Giuseppe Palomba, Negine Paul, Akshant Pathak, Giovanna Pavone, Rostislav Pavlov, Raul Pinillla, Omeed Rasheed, Anouar Remini, Andrey Ryabov, Elgun Samadov, Inian Samarasam, Erdene Sandag, Jorge Santos, Elio Sanchez, Azize Saroglu, Dimitrios Schizas, Pedro Azevedo Serralheiro, Oguzhan Simsek, Dmitry Sobolev, Amine Souadka, José Vieira de Sousa, Fabiana Sousa, Muhammed Suer, Suraj Surendran, Athanasios Syllaios, Aamir Ali Syed, Daiki Terajima, Merve Tokocin, Tania Triantafyllou, Server Uludağ, Tevfik Uprak, Susan Vaz, Massimo Vecchiato, Georgios Ioannis Verras, Massimiliano Veroux, Kelvin Voon, Kirill Vovin, Myla Yacob, Maciej Walędziak, Alexander Zacharenko, Fatima Tu Zahara, Rishi Singhal, Kamal Mahawar, HOLD Study Collaborative Group, Ahmed M. Abbas, Imed Abbassi, Ibrahim Abdelhamid, Federico Abente, Haider Abid, Ayman Abouleid, Hans Débora Acín-Gándara, Ademola A. Adeyeye, Alaa Mohamed Ads, Nezih Akkapulu, Talar Vartanoglu Aktokmakyan, Sergei Aksenov, Peter Ikponmwosa Agbonrofo, Mosheer S. Al-Dahdouh, Nigar Allahverdiyeva, Manar Al-Shami, Zaid Al-Sheikh Ali, Rawan Mohammed Al-Sallal, Ahmad Malek Alsheikh, Hamza Al-Naggar, Aiman Allawgalli, Subhi Zahi Al-Issawi, Sief-Addeen Al-Tahayneh, Michele Ammendola, Ahmed Y. Ammar, Laila Amrani, Mario Annecchiarico, Elissavet Anestiadou, Luciano Antozzi, Giovanni Aprea, Giulio Argenio, Akif Enes Arikan, Alan Askari, Alberto Assisi, Nora Ra’ed Atiah, Selmy S. Awad, Karim Ayed, Kemal Arslan, Jean-Baptiste Bachet, Juan Balcazar, Vignesh Bala, Guillermo Ponce De Leon Ballesteros, Christina D. Bali, Andrea Balla, Laura E. R. Barbosa, Stefano Bartolomeo, Raffaele Basile, Nurur Bayramov, Aparna Govil Bhasker, Usman M. Bello, Zakaria Houssain Belkhadir, Mahesh Bendemagal, Amine Benkabbou, Solomon Bera, Antonio Jose Bernardes, Patrícia Bernardo, Federica Bianco, Stefanos Bitsianis, Joana S. Bolota, Alessandro Bonomi, Nuno Borges, Haktan Övül Bozkır, El Ahmadi Brahim, Vittorio Bresadola, Marcos Bruna, Andrew D. Bueno, Virginia Cano Busnelli, Rasika Bulathsinhela, Francisco Cabral, Marcello Calabrò, Emir Capkinoglu, Marianna Capuano, Constantino Fondevila Campo, Salvatore Carrabetta, Clifford Caruana, Rui Casaca, Federico Del Castillo-Díez, Fausto Catena, Danilo Centonze, Anna Chaika, Alexandros Charalabopoulos, Aishwarya Chokshi, Lynn Chong, Christian Cotsoglou, Daniel M. Chircop, Sharfuddin Chowdhury, Beatriz Chumbinho, Sudhakar Chandran, Leonardo Luca Chiarella, Ivana Conversano, Paulo M. Costa, Gangalakshmi Chokkalingam, Liliana Cuevas, Jan Giuseppe Currò, Noureddine Chadli, Vladica V. Ćuk, Giovanni Dapri, Dragomir Dardanov, Anuj Dash, Gabriel Mihail Dimofte, Andrew R. Davies, Angel Diaz, Agron Dogjani, Onur Dülgeroğlu, Agustin Duro, Anne Sophie Dulac, Mohammed Hisham Eid, Hosam Elghadban, Nagm Eldin Abu Elnga, Mohamad Elfawal, Hazebroek Eric, Giuseppe Evola, Sarah K. Fahmi, Alice Filippelli, Yuri Fishman, Muratcan Fırat, Matthew Forshaw, Aakash Fouzdar, Francesco Frattini, Joana R. Frazão, Lewis S. Gall, Luca Casingena Garcia, Senthilkumar Ganesan, Ioannis Gerogiannis, Mohamed Hedi Ghalloussi, Elliot Goodman, James A. Gossage, Stefano Granieri, Amit Gupta, Ashok R. Gunawardene, Rahul Gupta, Hytham Hamid, Abdul Rahman Hammadieh, Joshua N. Hammerschlag, Francisco J. Henao, Rufino Eduardo Herrera, Michael W. Hii, Samir Hosny, Joshua Hong, Nikolaos Intzes, Orestis Ioannidis, Omorodion Omoruyi Irowa, Ivaylo Ivanov, Rawia Jamal, Periyathambi Jambulingam, Mustafa A. Abu Jayyab, Alexander Julianov, Tatyana S. Kalinina, Aziz Kamalov, Ahmad Amir H. Kayali, Mohd Kayyal, Vijay Kumar, Mandeep Kaur, Askar J. Keen, Mark Kelly, Mohammad Kermansaravi, Guowei Kim, Michal Kisielewski, Mohamad Klib, Archana Khanduri, Mansoor Khan, Cheng Jing Khaw, Malak Khaled Khlouf, Karina Koreshkova, Bojan M. Kovačević, Patricia M. P. Lages, Oumaima Lahnaoui, Larry Weng Hong Lai, Pedro Laranjo, Konstantinos Lasithiotakis, Lazarou Lazaros, Pasquale Lepiane, Georgios D. Lianos, Alessio Licciardello, Corrado Da Lio, Gordon Liu, Sorinel Lunca, Andrea-Pierre Luzzi, Daniele Macchini, Annalisa Mancin, Mário S. Marcos, Rita V. Martins, Alexander Mahendran, Mohammed Anass Majbar, Marina Makurina, Rosa J. Matias, Mustafa Sabri Massadi, Ruqaya Masri, Jean Mbonicura, Isabel M. Mesquita, Milica A. Milentijević, Migena Miza, Lourenço Moniz, Paolo Morgagni, Federico Moser, Adnan Mohammed, Raouf Mohsine, Cecília Monteiro, Isabella Mondi, Daniele Morezzi, Ana-Maria Musina, Areej Esam Nadeesh, Carlo Nagliati, Eddy Lincango Naranjo, Ayman Ibrahim Naser, Pueya Nashidengo, Aleksandr Neimark, Ionut Negoi, Prabhu N. Nesargikar, Chiara Nessi, Elena Herranz Van Nood, Lucio Rircardo Obeide, Stefano Olmi, Joana Ip Oliveira, Susana S. Onofre, Steve-Nation Nehiweze Oriakhi, Mouaqit Ouadii, Mehmet Faik Özçelik, Zeynep Özdemir, Mahir Ozmen, Giovanni Domenico De Palma, Anshuman Pandey, Hugo Palmieri, Bernardo L. Patrício, Caghan Peksen, Plamen Petkov, Enrico Pinotti, Gegam Pogosyan, Sjaak Pouwels, Alexandra Pupo, Aisha Qayum, Carlos Coello Quan, Paulo Ramos, Deepak Rajput, Shiv Rajan, Muhammad Shahan Raza, Matthew Read, Sofia Reis, Samuel E. Rey, João G. Ribeiro, Carolina Righetti, Cristian-Ene Roata, Emanuele Romairone, Homayoon Roshan, Laura Rosner, Elena Ruiz-Úcar, Antonio Santangelo, Catarina Rolo Santos, Amira Said, Alpaslan Şahin, Nasser Sakran, Kamil Salimzyanov, Federica Saraceno, Hitesh Sarda, Osman Anil Savas, Marah Ahmad Sawaftah, Mohammad Ahmad Sawaftah, Khaled Ahmad Sawaftah, Ahsan Shafiq, Azhar Shabbir, Amir Shariff, Franco Signorini, Theodoros A. Sidiropoulos, Silvia Silva, Jimmy Bok Yan So, João Pinto De Sousa, Paulo A. Soares, Leonardo Solaini, Eleftherios Spartalis, Michael Spartalis, Konstantinos Stratoulias, Duminda Subasinghe, Shankar Subbarayan, Aziz Sumer, Fjorenta Sulo, Savvas Symeonidis, Kimutai Ronoh Sylvester, Safwan Taha, Flavio Roberto Takeda, Athanasios Tampakis, Nicola Tartaglia, Abdallah R. Temerik, Nilanjana Tewari, Anisse Tidjane, Omar Tilioua, Charalampos Theodoropoulos, Aleš Tomažič, Carlos Toro-Huamanchumo, Alexandra Triantafyllou, Ioannis Tsikritzakis, Tyseer Median Tyseer, Fabio Tuminello, Fara Uccelli, Matteo Uccelli, Mehmet Eşref Ulutaş, Cihan Uras, Anna Utkina, Michail Vailas, Jean Christophe Vaillant, José I. Valenzuela, Carlo Vallicelli, Natalia Velenciuc, Daunia Verdi, Marlon A. Vesga, João S. Vieira, Nakul Viswanath Ivan Vlahovic, Samuel Wanjara, Ishaan Wazir, Zhuoqi Wei, Cacio Wietzycoski, Wojciech M. Wysocki, Bara’ Sami Yacoub, Wah Yang, Raghad Yousef Yassin, Ayşegül Yılmaz, David Yordanov, Kağan Zengin, Valentina Zucchini, Mauricio Zuluaga

**Affiliations:** 1 NOVA Medical School – Faculdade de Ciências Médicas, Universidade Nova de Lisboa | ULS São Jose, Campo Dos Mártires da Pátria 130, 1169-056 Lisbon, Portugal; 2 Department of surgery and Cancer. Imperial College, London, UK; 3 Department of Surgery, National Cancer Institute, Cairo University, Giza, Egypt; 4 Royal Adelaide Hospital, Adelaide, South Australia Australia; 5 Power Stats Statistical Consulting, West Ryde, Australia; 6 James Cook University Hospital, Cleveland, UK; 7 University Hospitals of Leicester, Leicester, UK; 8 National Centre for Oesophageal and Gastric Cancer, Trinity St James’ Cancer Institute, St James’ Hospital Clinical Senior Lecturer, Trinity College Dublin, Dublin, Ireland; 9 Advocate Illinois Masonic Medical Center, 836 W Wellington AVE, Chicago, IL 60657 USA; 10 Research Scholar in the Department of Surgery, University of Illinois at Chicago, Chicago, IL 60657 USA; 11 University Hospitals Coventry & Warwickshire, Coventry, UK; 12 P. Hertsen Moscow Oncology Research Institute, Moscow, Russia; 13 National Ribat University, Khartoum, Sudan; 14 Department of Gastroenterological Surgery, Dicle University School of Medicine, Diyarbakir, Turkey; 15 Al-Basheer Hospital, Amman, Jordan; 16 21 September University, Sana’a, Yemen; 17 ULS Coimbra PT, Coimbra, Portugal; 18 Ibn Al-Nafees Hospital, Damascus, Syria; 19 Patel Hospital, Karachi, Pakistan; 20 Hospital Privado Universitario de Córdoba, Córdoba, Argentina; 21 Shaukat Khanum Memorial Cancer Hospital and Research Centre, Lahore, Pakistan; 22 Faculty of Medicine, Ain-Shams University, Cairo, Egypt; 23 Guy’s and St Thomas’ NHS Foundation Trust, London, UK; 24 Habib Bougatfa Hospital, Bizerte, Tunisia; 25 RISE-Health, Departamento de Cirurgia E Fisiologia, Faculdade de Medicina, Universidade Do Porto, Alameda Prof. Hernâni Monteiro, 4200-319 Porto, Portugal | ULSSJoao, Alameda Prof. Hernâni Monteiro, 4200-319 Porto, Portugal; 26 Tishreen University Hospital, Laktakia, Syria; 27 Clinical Hospital Center Osijek, Osijek, Croatia; 28 Instituto Português de Oncologia de Lisboa Francisco Gentil, Lisbon, Portugal; 29 Bakirkoy Dr. Sadi Konuk Research and Training Hospital, Istanbul, Turkey; 30 Alma Mater Studiorum - Università Di Bologna, Bologna, Italy; 31 Lorenzo Bonomo Hospital, Andria, Italy; 32 Department of General Surgery, Marmara University, Istanbul, Turkey; 33 Athens Medical Center, Athens, Greece; 34 University of Samsun, Samsun Training and Research Hospital, Samsun, Turkey; 35 Centro Hospitalar Universitário Porto-Hospital Santo António, Porto, Portugal; 36 Saint-Petersburg State University Hospital, St. Petersburg, Russia; 37 General and Oncologic Surgery, Morgagni – Pierantoni Hospital, Forlì, Italy; 38 Siberian State Medical University, Tomsk, Russia; 39 Mongolian National University of Medical Sciences, Ulaanbaatar, Mongolia; 40 Istanbul University Cerrahpasa - Cerrahpasa School of Medicine, Istanbul, Turkey; 41 Immanuel Kant Baltic Federal University, Kaliningrad, Russia; 42 Hospital Prof. Doutor Fernando Fonseca, Amadora, Portugal; 43 Leyla Medical Center, Baku, Azerbaijan; 44 Department of Hepato-Biliary and Pancreatic Surgery, Sorbonne Université, Assistance Publique- Hôpitaux de Paris, AP-HP, Pitié-Salpêtrière University Hospital, Paris, France; 45 Pavlov First Saint Petersburg State Medical University, Saint Petersburg, Russia; 46 University Medical Centre Ljubljana, Ljubljana, Slovenia; 47 Instituto Nacional de Cancerología, Bogotá, Colombia; 48 University Of Health Science Konya City Hospital, Konya, Turkey; 49 Hospital de Gastroenterología Dr. Bonorino Udaondo, Ciudad Autónoma de Buenos Aires, Argentina; 50 National Cancer Center Hospital East, Kashiwa, Japan; 51 University of Glasgow, Glasgow, Scotland; 52 Medical University of Silesia, Katowice, Poland; 53 Statistical Consulting, NOVA Information Management School, Universidade Nova de Lisboa, Carcavelos, Portugal; 54 General Surgery Department, Hadassah Medical Organization and Faculty of Medicine, Hebrew University of Jerusalem, Jerusalem, Israel; 55 Centro Hospitalar Trás-Os-Montes E Alto Douro, Vila Real, Portugal; 56 University Oran 1/1St November 1954 University Hospital / The Algerian Laboratory of Research in Innovative Technologies Medicine (LARTIM), Oran, Algeria; 57 Bagcilar Training and Research Hospital, Istanbul, Turkey; 58 General University Hospital of Patras, Patras, Greece; 59 E. Agnelli Hospital, Pinerolo, Italy; 60 Azerbaijan Medical University, Nan, Azerbaijan; 61 Università Degli Studi Di Napoli, Federico II, Naples, Italy; 62 Christian Medical College Hospital, Vellore, India; 63 Delhi State Cancer Institute, Delhi, India; 64 University of Foggia, Foggia, Italy; 65 Instituto Nacional de Cancerología y Universidad Nacional de Colombia, Bogotá, Colombia; 66 Surgical Oncology Unit, Hospital Universitario San Ignacio and Pontificia Universidad Javeriana, Bogotá, Colombia; 67 Trakia Hospital, Stara Zagora, Bulgaria; 68 First Department of Surgery, National and Kapodistrian University of Athens, Athens, Greece; 69 Mohammed 5, University in Rabat National Institute of Oncology, Rabat, Morocco; 70 Centro Hospitalar Universitário de São João, Porto, Portugal; 71 Hacettepe University, Ankara, Turkey; 72 Hippocration General Hospital of Athens, Athens, Greece; 73 SOC Chirurgia Generale, Udine, Italy; 74 Azienda Policlinico San Marco, University of Catania, Catania, Italy; 75 Penang General Hospital, Penang, Malaysia; 76 Military Institute of Medicine, Warsaw, Poland; 77 University Hospital Birmingham NHS Foundation Trust, Birmingham, UK; 78 South Tyneside and Sunderland NHS Foundation Trust, University of Sunderland, Sunderland, UK

**Keywords:** Gastric cancer, Elective surgery, Morbidity and mortality, 90-Day postoperative outcomes, Multinational audit, Surgical Complications, Anastomotic leaks, Patient safety

## Abstract

**Background:**

Data on multinational 90-day mortality and morbidity rates after surgery for gastric cancer is limited in the literature. This study aimed to understand the 90-day mortality and morbidity outcomes among patients undergoing elective gastric cancer surgery, as in the GASTRODATA Registry, and to identify associated risk factors.

**Methods:**

We conducted an international prospective study on patients aged ≥ 18 years undergoing elective surgery for gastric cancer with curative intent from January 4 to September 30, 2022. Known metastatic disease, concurrent secondary cancers, gastrointestinal stromal tumour (GIST) and Siewert type I/II oesophagogastric junction malignancies were excluded. Univariate and multivariate logistic regression were used to identify variables associated with the 90-day outcome.

**Results:**

380 collaborators from 47 countries submitted data on 1538 patients. Median age was 65 years (IQR: 19–94), and 58.5% were males. 90-day morbidity and mortality rates were 38.2% (n = 587) and 2.9% (n = 45), respectively. Pre-operative higher Charlson Comorbidity Index, higher ASA score, pre-operative weight loss > 10%, positive specimen margin, and post-operative pathological IV staging (p value < 0.05) were significantly associated with clinically relevant complications and mortality.

**Conclusion:**

Elective gastric cancer surgery has a 90-day morbidity of 38.2% and a 90-day mortality of 2.9% globally. This study provided the most comprehensive international 90-day prospective data to date regarding gastric cancer surgery. Several factors associated with higher morbidity were identified, highlighting the importance of a unified language on surgical morbidity, prehabilitation, and ongoing audits to enhance patient outcomes.

**Supplementary Information:**

The online version contains supplementary material available at 10.1007/s00423-025-03890-7.

## Introduction

Gastric cancer is the fifth most common malignancy and the fifth leading cause of cancer mortality worldwide, as reported by GLOBOCAN (Global Cancer Observatory) [1]. Surgical resection with regional lymphadenectomy remains the gold standard of treatment [2–4]. The primary aim of treatment is complete resection with microscopically negative margins. While subtotal gastrectomy is favoured to reduce postoperative morbidity, total gastrectomy (TG) is often necessary [5, 6] to achieve adequate treatment.

In the management of gastric cancer, the shift towards evidence-based and precision-oriented care has led to the adoption of standardised treatment protocols [4, 7] and improved surgical performance. This includes the integration of multimodal strategies, minimally invasive surgical techniques, and the development of comprehensive complication registries and follow-up databases [2, 8]. The centralisation of surgical procedures and the adoption of multimodal treatment strategies have improved outcomes of gastric cancer surgery [7, 9] by targeting both local and systemic disease, enhancing surgical effectiveness and supporting patient recovery. Delivered within a multidisciplinary framework, these approaches optimise both oncological results and perioperative recovery. Nevertheless, morbidity and mortality rates vary significantly among countries [10, 11]. This can be attributed to several factors, including screening programmes, disease incidence, management protocols, stage of cancer at presentation, surgical techniques, and follow-up [12].

The mortality rates in the East are consistently lower [13, 14], with 30-day mortality rates below 1% [15, 16]. Conversely, western countries report a wider morbidity range [17, 18] from 6.3% to 43% [13, 19, 20] and higher mortality rates, spanning 2 to 12.8% [21–23], mostly reported until 30 days postoperatively.

Complications following surgical intervention in gastric cancer patients adversely influence the oncological prognosis, manifesting in reduced survival rates [24] and earlier recurrence. While several studies have reported on 60- and 90-day postoperative morbidity and mortality in gastric cancer surgery [9, 14, 25–29], many have been limited by a focus on a single surgical technique, a single-country dataset, or relatively small multicentre cohorts [9, 14, 25, 26]. Our study contributes to this body of work by providing the most comprehensive 90-day prospective data to date, encompassing large-scale, international, prospective data across various surgical approaches.

The main aims of this study were to understand the global 90-day morbidity and mortality rates of curative gastric cancer surgery and to understand factors associated with morbidity.

## Methods

### Study Design

We conducted a global, multicentre, prospective observational cohort study. The study was registered as a clinical audit at James Cook University Hospital, Middlesbrough, UK (Registration Number CATS ID 8504). The primary aim was to assess the 90-day mortality and morbidity rates of patients who underwent elective gastric cancer surgery between 01/04/2022 and 30/09/2022 at participating centres. The secondary aim was to identify the factors influencing these rates.

### Data collection

We collected demographic data, pre- operative data (cTNM staging, neoadjuvant therapy), surgical data (gastric resection and lymphadenectomy, surgical approach, reconstruction, jejunostomy, surgery duration and definitive histopathology report), and 90-day outcomes. The Gastrectomy Complications Consensus Group (GCCG) of GASTRODATA [27] was used to identify postoperative complications and morbidity. General postoperative complications included systemic events such as infections, respiratory or renal failure, while surgical postoperative complications referred to events directly related to the operative site, such as anastomotic leaks, postoperative bleeding, or bowel perforation. We used the Clavien-Dindo (CD) classification [30, 31] to record the severity of complications, including mortality. We defined a clinically relevant morbidity as CD ≥ II [32]. This study focused on the number of patients who developed complications rather than the number of complications. The highest CD score was considered when a patient experienced more than one complication. The eighth edition of the TNM (TNM-8) was used for tumour staging [33].

The study was promoted to the members of *The Upper Gastrointestinal Surgical Society* (TUGSS) through emails and widely disseminated on social media. Researchers collected anonymised patient data and submitted it in a password-protected Microsoft Excel sheet and the password was shared separately to preserve data confidentiality. A *'Data Dictionary'* file was shared with all researchers, Appendix 1*.* To mitigate selection bias, collaborators were asked to record all consecutive eligible patients during the study period. After submission, we examined the data for incomplete or missing information and contacted collaborators for clarification as needed.

### Participants

All adults (≥ 18 years) diagnosed with primary gastric cancer, including Siewert type III, who were candidates for elective surgery with curative intent. Exclusion criteria were patients with gastrointestinal stromal tumour (GIST), preoperative known metastatic disease, positive peritoneal cytology at staging laparoscopy or definitive surgery, concurrent secondary cancers, those with Siewert type I or II oesophagogastric junction malignancies, and patients requiring emergency surgical interventions or other surgeries in addition to the primary gastric malignancy treatment.

### Statistical analysis

Univariate and multivariate logistic regression were used to identify variables associated with minor (Clavien-Dindo 0 and I) and clinically significant (Clavien-Dindo II-V) postoperative outcomes. Multivariable analysis was performed for factors with a p-value of ≤ 0.20 in the univariable analysis. All statistical testing was two‐sided, with significance defined as a *p*-value < 0.05. Lasso analysis for logistic regression was performed to validate the findings. The dataset was split into a testing sample (75%) and a validation sample (25%) with random allocation. Lasso analysis for logistic regression was performed to validate and further explore the relevance of the variables selected by the multivariate logistic regression model. Data management and statistical analyses were performed using Stata® SE for Windows® version 15.1 (StataCorp, College Station, TX, USA).

### Ethical considerations

Collaborators were responsible for obtaining local permission and patient consent and documenting that conversation in the patient record.

## Results

### Pre-operative demographic, clinical, and imaging data

**Fig. 1:**
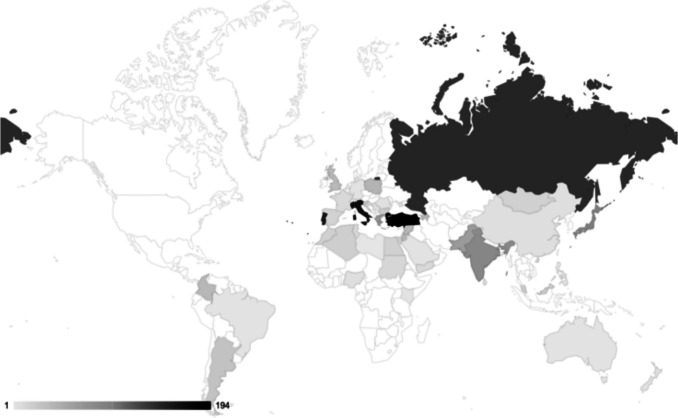
Heatmap Patients/Country: Number of patients (n = 1538) included in the HOLD Study by country (47 countries)

The study considered data from 1,670 patients across 138 hospitals in 47 countries, submitted by 380 collaborators (Fig. 1). We excluded 132 (7.9%) patients as they did not meet our inclusion criteria, resulting in a final analysis of 1,538 patients. Table 1 shows the demographic characteristics of our cohort.

**Table 1:** Basic demographic data (n = 1538)

PATIENT CHARACTERISTICS		GASTRIC RESECTION					
		Overall, N = 1538	Subtotal Gastrectomy, N = 761	Total Gastrectomy, N = 627	Extended Total Gastrectomy, N = 74	Proximal Gastrectomy, N = 48	Oesophago-Gastrectomy, N = 28
Age		64,2 ± 13,4	65,9 ±12,9	62,6 ± 13,5	60,9 ± 15,4	66,2 ± 11,3	58,5 ± 13,5
Gender | Male		966 (62,8%)	441 (28,7%)	418 (27,2%)	51 (3,3%)	35 (2,3%)	21 (1,4%)
Preoperative BMI		24,7 ± 4,7	24,4 ± 4,6	24,9 ± 4,9	25,7 ± 4,4	26,5 ± 5,8	23,9 ± 3,4
Preoperative Weight Loss > 10%		624 (40,6%)	295 (19,2%)	267 (17,4%)	32 (2,1%)	14 (0,9%)	16 (1,0%)
ASA	1	210 (13,7%)	100 (6,5%)	94(6,1%)	7 (0,5%)	3 (0,2%)	6 (0,4%)
	2	742 (48,2%)	372(24,2%)	298 (19,4%)	33 (2,1%)	29 (1,9%)	10 (0,7%)
	3	551 (35,8%)	275 (17,9%)	216 (14,0%)	33 (2,1%)	15 (1,0%)	12 (0,8%)
	4	35 (2,3%)	14 (0,9%)	19 (1,2%)	1 (0,1%)	1 (0,1%)	-
Charlson Comorbidity index (CCI)		4,8 ± 2,3	4,9 ± 1,6	4,7 ± 2,1	5,9 ± 2,4	4,9 ± 3,0	5,1 ±2,9
ECOG (a)	0	686 (44,6%)	358 (23,3%)	266 (17,3%)	35 (2,3%)	15 (1,0%)	12 (0,8%)
	1	548 (35,7%)	258 (16,8%)	241 (15,7%)	20 (1,3%)	18 (1,2%)	11 (0,7%)
	2	206 (13,4%)	100 (6,5%)	83 (5,4%)	10 (0,7%)	10 (0,7%)	3 (0,2%)
	3	84 (5,5%)	41 (2,7%)	30 (2,0%)	6 (0,4%)	5 (0,3%)	2 (0,1%)
	4	13 (0,8%)	4 (0,3%)	6 (0,4%)	3 (0,2%)	-	-

*BMI* Body Mass Index; *ASA* American Society of Anaesthesiologists; *ECOG* Eastern Cooperative Oncology Group Performance Status;

(a) In *ECOG*, information was missing from 1 patient, a Total gastrectomy

The median age of patients was 65 years (interquartile Range -IQR: 19–94), with 50% of patients aged between 56 and 74 years. Overall, 62.8% were male. In our study, each additional year of age was correlated with a 1.3% increase in the odds (p = 0.0013) of experiencing clinically relevant 90-day morbidity (CD II-V) and mortality. The median body mass index (BMI) was 24.5 kg/m^2^, and 40.6% of the patients experienced severe weight loss (> 10% of body weight) in the 6 months preceding surgery.

Caucasians accounted for 58.5% of all patients, followed by Asians (26.0%). Nearly half of the cohort (48.2%) had an American Society of Anaesthesiologists (ASA) score of II, and 44.6% had an Eastern Cooperative Oncology Group (ECOG) Performance Status [34] of 0. Approximately 52.7% of the patients had a Charlson Comorbidity Index (CCI) score of 5 or higher [35, 36].

More than half (61.5%) were diagnosed as cT2-T3 stages with nodal (cN +) involvement (51.7%), and 4.4% were suspected to have cT4 disease. Pre-operatively, 3.4% of patients had incomplete staging [2–4, 33] and 11.2% had unknown histology [2–4]. Despite the high prevalence of advanced gastric cancer at diagnosis, fewer than half of patients received neoadjuvant therapy. Among those staged as cT3–cT4, 57.1% underwent neoadjuvant chemotherapy, accounting for 76.0% of all patients treated in the neoadjuvant setting. In total, 680 patients received neoadjuvant chemotherapy, predominantly those with cT3–cT4 disease (n = 504) and/or node-positive disease (cN + , n = 481). Treatment completion, however, was suboptimal, with 13.7% (n = 93) failing to complete the planned regimen. Notably, completion rates were markedly lower in patients aged > 70 years (28.3%) when compared with younger patients (54.4%). Patients in the "No neoadjuvant chemotherapy but should" subgroup showed higher Charlson Comorbidity Index scores, indicating a greater burden of co-existing conditions and suggesting increased complexity and risk in their surgical care. Table 2 provides details of staging and neoadjuvant therapy.

**Table 2:** Pre-operative Staging and Oncological Data (n = 1538)

PATIENT CHARACTERISTICS		GASTRIC RESECTION					
		Overall, N = 1538	Subtotal Gastrectomy, N = 761	Total Gastrectomy, N = 627	Extended Total Gastrectomy, N = 74	Proximal Gastrectomy, N = 48	Oesophago-Gastrectomy, N = 28
cT(a)	T0	**10 (0,7%)**	3 (0,2%)	5 (0,3%)	1 (0,1%)	1 (0,1%)	-
	T1	**206 (13,4%)**	134 (8,7%)	48 (3,1%)	2 (0,1%)	18 (1,2%)	4 (0,3%)
	T2	**334 (21,7%)**	181 (11,8%)	141 (9,2%)	4 (0,3%)	5 (0,3%)	3 (0,2%)
	T3	**612 (39,8%)**	270 (17,6%)	281 (18,3%)	40 (2,6%)	10 (0,7%)	11 (0,7%)
	T4	**271 (17,6%)**	122 (7,9%)	117 (7,6%)	21 (1,4%)	5 (0,3%)	6 (0,4%)
	Tis	**4 (0,3%)**	3 (0,2%)	1 (0,1%)	-	-	-
	Tx	**101 (6,6%)**	48 (3,1%)	34 (2,2%)	6 (0,4%)	9 (0,6%)	4 (0,3%)
cN(a)	N0	**586 (38,1%)**	337 (21,9%)	204 (13,3%)	16 (1,0%)	22 (1,4%)	7 (0,5%)
	N +	**795 (51,7%)**	346 (22,5%)	364 (23,7%)	53 (3,4%)	16 (1,0%)	16 (1,0%)
	Nx	**157 (10,2%)**	78 (5,1%)	59 (3,8%)	5 (0,3%)	10 (0,7%)	5 (0,3%)
cM(a)	M0	**1366 (88,8%)**	698 (45,4%)	560 (36,4%)	51 (3,3%)	36 (2,3%)	21 (1,4%)
	M1	**68 (4,4%)**	24 (1,6%)	25 (1,6%)	17 (1,1%)	1 (0,1%)	1 (0,1%)
	Mx	**104 (6,8%)**	39 (2,5%)	42 (2,7%)	6 (0,4%)	11 (0,7%)	6 (0,4%)
Neoadjuvant Chemotherapy	Given, completed	**587 (38,2%)**	223 (14,5%)	293 (19,1%)	44 (2,9%)	13 (0,8%)	14 (0,9%)
	Given, not completed	**93 (6,1%)**	28 (1,8%)	44 (2,9%)	14 (0,9%)	5 (0,3%)	2 (0,1%)
	Not given	**856 (55,7%)**	509 (33,1%)	289 (18,8%)	16 (1,0%)	30 (2,0%)	12 (0,8%)
	Missing data	**2 (0,1%)**	1 (0,1%)	1 (0,1%)	-	-	-

(a) James D. Brierley (Editor) MKG (Editor), CW (Editor). TNM Classification of Malignant Tumours, 8th Edition. Wiley-Blackwell; 2017

Gastric adenocarcinoma (87.7%) was the most common type, followed by adenosquamous carcinoma (2.9%) and undifferentiated carcinoma (2.1%) [37]. Other neoplasms, such as squamous cell carcinoma and lymphoma, accounted for 1.6% and 4.4% of the cases, respectively.

### Surgical approach and postoperative histopathology

Subtotal (49.5%, n = 761) and total (40.8%, n = 627) gastrectomies were the most common types of surgery, as shown in Table 3. These resections presented a 40% lower risk (p < 0.001) of clinically relevant postoperative morbidity and mortality when compared to all other resections. Total gastrectomy had the highest conversion to open rate (13.2%). Only a minority of conversions to open (12.3%) were due to intraoperative adverse events.

**Table 3:** Surgery details regarding patients undergoing elective gastric surgery for malignancy (n = 1538)

PATIENT CHARACTERISTICS		GASTRIC RESECTION					
		Overall, N = 1538	Subtotal Gastrectomy, N = 761	Total Gastrectomy, N = 627	Extended Total Gastrectomy, N = 74	Proximal Gastrectomy, N = 48	Oesophago-Gastrectomy, N = 28
Surgical Approach	Open	**874 (56,8%)**	408 (26,5%)	378 (24,6%)	42 (2,7%)	29 (1,9%)	17 (1,1%)
	Laparoscopic	**452 (29,4%)**	266 (17,3%)	145 (9,4%)	20 (1,3%)	14 (0,9%)	7 (0,5%)
	Robotic	**69 (4,5%)**	39 (2,5%)	21 (1,4%)	2 (0,1%)	5 (0,3%)	2 (0,1%)
	Laparoscopic/Robotic Converted to Open	**143 (9,3%)**	48 (3,1%)	83 (5,4%)	10 (0,7%)	-	2 (0,1%)
Reconstruction	Roux en Y	**1113 (72,4%)**	471 (30,6%)	564 (36,7%)	57 (3,7%)	8 (0,5%)	13 (0,8%)
	Bilroth I	**27 (1,8%)**	21 (1,4%)	2 (0,1%)	1 (0,1%)	3 (0,2%)	-
	Bilroth II	**285 (18,5%)**	248 (16,1%)	21 (1,4%)	8 (0,5%)	2 (0,1%)	6 (0,4%)
	Jejunal Interposition	**34 (2,2%)**	6 (0,4%)	16 (1,0%)	6 (0,4%)	4 (0,3%)	2 (0,1%)
	Colonic Interposition	**9 (0,6%)**	1 (0,1%)	1 (0,1%)	1 (0,1%)	-	6 (0,4%)
	Gastric Reconstruction	**30 (2,0%)**	5 (0,3%)	2 (0,1%)	1 (0,1%)	21 (1,4%)	1 (0,1%)
	Undetailed Reconstruction	**40 (2,6%)**	9 (0,6%)	21 (1,4%)	-	10 (0,7%)	-
Jejunostomy performed		**169 (11,0%)**	55 (3,6%)	84 (5,5%)	14 (0,9%)	4 (0,3%)	12 (0,8%)
Lympha-denectomy	D0	**31 (2,0%)**	18 (1,2%)	7 (0,5%)	2 (0,1%)	4 (0,3%)	-
	D1	**258 (16,8%)**	131 (8,5%)	95 (6,2%)	14 (0,9%)	8 (0,5%)	10 (0,7%)
	D1 +	**216 (14,0%)**	125 (8,1%)	68 (4,4%)	8 (0,5%)	14 (0,9%)	1 (0,1%)
	D2	**970 (63,1%)**	470 (30,6%)	420 (27,3%)	43 (2,8%)	21 (1,4%)	16 (1,0%)
	D2 +	**62 (4,0%)**	16 (1,0%)	37 (2,4%)	7 (0,5%)	1 (0,1%)	1 (0,1%)
	Missing data	**1 (0,1%)**	1 (0,1%)	-	-	-	-
Lymph nodes harvested in D2 (average ± standard deviation)		**28,6 **±**14,5**	29,5 ±14,9	28,3 ±14,4	27,9 ±11,3	19,2 ±8,1	25,7 ±10,7

Open surgery (56.8%) was the most common technique, followed by laparoscopic surgery (29.4%) and robotic surgery (4.5%). Laparoscopic surgery was more common in early stages (49.2% in Ia), while open surgery was the preferred technique in advanced stages (80.9% in IIIc). Roux-en-Y reconstruction was the primary approach for gastrointestinal tract reconstruction (72.4%), and a complete macroscopic resection margin was achieved in 89.1% (n = 1371) of patients. The median duration of surgery was 220.0 min (IQR: 161.3–270.0).

D2 lymphadenectomy was reported in 63.1% (n = 970) of patients, with a median of 22.5 lymph nodes (IQR: 14.0–33.5) harvested. Adequate lymph node retrieval (> 15 lymph nodes) for pathological staging was achieved in 79.1% of cases, with 5.2% missing data. Jejunostomy was performed in 11.0% of the cohort (n = 169), most commonly in oesophago-gastric resections.

The final pathology report (pTNM) was completed in 94.7% (n = 1456) of patients, with 74.2% (n = 1141) staged as locally advanced gastric cancer (≥ Ib) (Table 4). Node-negative status (pN0) was observed in 38.6% of the patients. Chemotherapy details were inconsistently reported across centres, limiting treatment-specific analysis. The majority of patients received standard perioperative regimens (e.g., FLOT or ECF). Pathological complete remission (pT0) occurred in 4.3% of patients receiving neoadjuvant chemotherapy (n=29). In definitive pathology, early gastric cancer was identified in 15.8% (n = 238) of cases, an increase from 13.4% identified at clinical staging.

**Table 4:** Data regarding definitive histopathology report after surgery (n = 1538)

PATIENT CHARACTERISTICS		GASTRIC RESECTION					
		Overall, N = 1538	Subtotal Gastrectomy, N = 761	Total Gastrectomy, N = 627	Extended Total Gastrectomy, N = 74	Proximal Gastrectomy, N = 48	Oesophago-Gastrectomy, N = 28
Tumour Histology (WHO) (a)	Adenocarcinoma	**1349 (87,7%)**	681 (44,3%)	554 (36,0%)	55 (3,6%)	43 (2,8%)	16 (1,0%)
	Adenosquamous	**45 (2,9%)**	18 (1,2%)	15 (1,0%)	6 (0,4%)	1 (0,1%)	5 (0,3%)
	Undifferentiated	**33 (2,1%)**	16 (1,0%)	12 (0,8%)	2 (0,1%)	1 (0,1%)	2 (0,1%)
	Squamous Cell Carcinoma	**24 (1,6%)**	10 (0,7%)	9 (0,6%)	1 (0,1%)	1 (0,1%)	3 (0,2%)
	Carcinoma with lymphoid stroma	**13 (0,8%)**	3 (0,2%)	6 (0,4%)	2 (0,1%)	1 (0,1%)	1 (0,1%)
	Hepatoid Carcinoma	**6 (0,4%)**	1 (0,1%)	1 (0,1%)	4 (0,3%)	-	-
	Other	**68 (4,4%)**	32 (2,1%)	30 (2,0%)	4 (0,3%)	1 (0,1%)	1 (0,1%)
pT	T0	**37 (2,4%)**	19 (1,2%)	16 (1,0%)	1 (0,1%)	-	1 (0,1%)
	Tis	**2 (0,1%)**	-	1 (0,1%)	1 (0,1%)	-	-
	T1	**324 (21,1%)**	201 (13,1%)	86 (5,6%)	6 (0,4%)	25 (1,6%)	6 (0,4%)
	T2	**269 (17,5%)**	142 (9,2%)	102 (6,6%)	11 (0,7%)	7 (0,5%)	7 (0,5%)
	T3	**527 (34,3%)**	225 (14,6%)	251(16,3%)	30 (2,0%)	11 (0,7%)	10 (0,7%)
	T4	**375 (24,4%)**	172 (11,2%)	169 (11,0%)	25 (1,6%)	5 (0,3%)	4 (0,3%)
	Tx	**4 (0,3%)**	2 (0,1%)	2 (0,1%)	-	-	-
pN	N0	**594 (38,6%)**	337 (21,9%)	207 (13,5%)	17 (1,1%)	25 (1,6%)	8 (0,5%)
	N1	**269 (17,5%)**	139 (9,0%)	102 (6,6%)	16 (1,0%)	11 (0,7%)	1 (0,1%)
	N2	**288 (18,7%)**	121 (7,9%)	123 (8,0%)	24 (1,6%)	8 (0,5%)	12 (0,8%)
	N3	**281 (24,8%)**	161 (10,5%)	192 (12,5%)	17 (1,1%)	4 (0,3%)	7 (0,4%)
	Nx	**6 (0,4%)**	3 (0,2%)	3 (0,2%)	-	-	-
Resection Margin	R0	**1371 (89,1%)**	707 (46,0%)	553 (36,0%)	50 (3,3%)	41 (2,7%)	20 (1,3%)
	R1	**130 (8,5%)**	47 (31%)	57 (3,7%)	16 (1,0%)	7 (0,5%)	3 (0,2%)
	R2	**37 (2,4%)**	7 (0,5%)	17 (1,1%)	8 (0,5%)	-	5 (0,3%)
Lymph nodes	> 15	**1217 (79,1%)**	35 (2,3%)	599 (38,9%)	506 (32,9%)	57 (3,7%)	20 (1,3%)
	Total Number (pN total)	**26,7** ± **15,0**	26,5 ±15,6	27,6 ± 14,8	26,2 ±13,5	20,1 ±11,9	24,7 ±10,9
	Positive (pN +)	**4,7** ± **7,1**	3,8 ±6,3	6,0 ±8,2	4,7 ±5,2	2,2 ±3,6	5,8 ±7,2

(a) Kushima R LGRM. WHO Classification of Tumours: Digestive Systemic Tumours. 5th ed. WHO Classification of Tumours Editorial Board, editor. Vol. Gastric Dysplasia. International Agency for Research on Cancer 2019

Less than half of the clinically suspected metastatic disease was confirmed as pM1 (i.e. peritoneal invasion or positive cytology), with 54.8% of pM1 also categorised as either R1 or R2 resection. Most patients (89.1%, n = 1371) achieved a microscopic R0 resection. R2 resections, which are rarely reported in gastric cancer surgery [38], were observed in 2.4% of cases. R1 resection was most frequent in patients undergoing extended total gastrectomy (21.6%) and in those with stage IIIa–IIIc disease (58.3%). Notably, approximately half of patients with R1 resections (54.5%) had received pre-operative chemotherapy.

### 90-day Morbidity and Mortality

In this cohort, the 90-day morbidity rate was 38.2% (n = 587), and the mortality rate was 2.9% (n = 45). Following Clavien Dindo (CD) classification, 12.0% (n = 184) had CD Grade I, 12.2% (n = 188) had CD Grade II, 10.5% (n = 162) had CD Grade III, and 3.4% (n = 53) had CD Grade IV (Table 5).

**Table 5:** Postoperative data of patients undergoing curative elective gastric surgery for malignancy (n = 1538)

90-DAY MORBIDITY and MORTALITY		GASTRIC RESECTION					
		Overall, N = 1538	Subtotal Gastrectomy, N = 761	Total Gastrectomy, N = 627	Extended Total Gastrectomy, N = 74	Proximal Gastrectomy, N = 48	Oesophago-Gastrectomy, N = 28
Clavien Dindo Class	I	**184 (12,0%)**	93 (6,0%)	76 (4,9%)	3 (0,2%)	7 (0,5%)	5 (0,3%)
	II	**188 (12,2%)**	90 (5,9%)	74 (4,8%)	13 (0,8%)	10 (0,7%)	1 (0,1%)
	IIIa	**85 (5,5%)**	38 (2,5%)	33 (2,1%)	8 (0,5%)	4 (0,3%)	2 (0,1%)
	IIIb	**77 (5,0%)**	40 (2,6%)	27 (1,8%)	7 (0,5%)	2 (0,1%)	1 (0,1%)
	IVa	**42 (2,7%)**	16 (1,0%)	17 (1,1%)	5 (0,3%)	1 (0,1%)	3 (0,2%)
	IVb	**11 (0,7%)**	3 (0,2%)	6 (0,4%)	1 (0,1%)	1 (0,1%)	-
	V (Mortality)	**45 (2,9%)**	22 (1,4%)	16 (1,0%)	5 (0,3%)	2 (0,1%)	-
	Total Morbidity and Mortality at 90-day	**632 (41,1%)**	302 (19,6%)	249 (16,2%)	42 (2,7%)	27 (1,8%)	12 (0,8%)
Postoperative Complications Incidence at 90 days (a)	Non-Surgical Infections (c)	**73 (4,7%)**	31(2,0%)	28 (1,8%)	2 (0,1%)	8 (0,5%)	4 (0,3%)
	Anastomotic leak (b)	**70 (4,6%)**	19 (1,2%)	37 (2,4%)	9 (0,6%)	3 (0,2%)	2 (0,1%)
	Abnormal fluid from drainage / abdominal collections WITHOUT Leak (b)	**52 (3,4%)**	30 (2,0%)	20 (1,3%)	1 (0,1%)	-	1 (0,1%)
	Postoperative bleeding requiring invasive treatment (b)	**40 (2,6%)**	16 (1,0%)	17 (1,1%)	5 (0,3%)	1 (0,1%)	1 (0,1%)
	Pleural effusion requiring drainage (c)	**36 (2,3%)**	11 (0,7%)	21 (1,4%)	2 (0,1%)	2 (0,1%)	-
	Postoperative bowel obstruction (b)	**34 (2,2%)**	14 (0,9%)	15 (1,0%)	3 (0,2%)	1 (0,1%)	1 (0,1%)
	Duodenal leak (b)	**29 (1,9%)**	20 (1,3%)	4 (0,3%)	4 (0,3%)	-	1 (0,1%)
	Respiratory failure requiring reintubation (c)	**21 (1,4%)**	10 (0,7%)	8 (0,5%)	2 (0,1%)	-	1 (0,1%)
	Need for prolonged intubation (> 24 h after surgery) OR Tracheostomy (c)	**21 (1,4%)**	4 (0,3%)	10 (0,7%)	5 (0,3%)	-	2 (0,1%)
	Pulmonary embolism (c)	**20 (1,3%)**	6 (0,4%)	10 (0,7%)	3 (0,2%)	1 (0,1%)	-
	Delayed gastric emptying (> 10th postoperative day) (b)	**18 (1,2%)**	14 (0,9%)	3 (0,2%)	-	1 (0,1%)	-
	Acute renal failure requiring CVVH/dialysis (c)	**15 (1,0%)**	8 (0,5%)	5 (0,3%)	2 (0,1%)	-	-
	Postoperative pancreatic fistula (b)	**14 (0,9%)**	7 (0,5%)	2 (0,1%)	4 (0,3%)		1 (0,1%)
	Myocardial infarction (c)	**14 (0,9%)**	6 (0,4%)	5 (0,3%)	1 (0,1%)	2 (0,1%)	-
	Postoperative bowel perforation or necrosis (b)	**9 (0,6%)**	3 (0,2%)	3 (0,2%)	3 (0,2%)	-	-
	Postoperative pancreatitis (b)	**7 (0,5%)**	2 (0,1%)	5 (0,3%)	-	-	-
	Others (d)	**296 (19,2%)**	146 (9,5%)	118 (7,7%)	17 (1,1%)	12 (0,8)	3 (0,2%)
Length of stay (days)		**11,0 **±**8,7**	10,5 ±8,6	11,0 ±8,7	15,2 ±11,3	11,6 ±5,6	12,6 ±6,5
Need for Reoperation		**124 (8,1%)**	68 (4,4%)	42 (2,7%)	10 (0,7%)	1 (0,1%)	3 (0,2%)
Readmission		**163 (10,6%)**	81 (5,3%)	59 (3,8%)	14 (0,9%)	5 (0,3%)	4 (0,3%)

(a) Following GASTRODATA Registry—Baiocchi GL, Giacopuzzi S, Marrelli D, Reim D, Piessen G, Matos da Costa P, et al. International consensus on a complications list after gastrectomy for cancer. Gastric Cancer. 2019 Jan 22;22(1):172–89

(b) Surgical Post Operative complications

(c) General Post Operative complications (non Surgical)

(d) Other included n = 9 Acute cardiac failure OR cardiac dysrhythmia, n = 8 Cardiopulmonary resuscitation, n = 3 acute liver dysfunction, n = 3 stroke with permanent deficit, n = 2 Pneumothorax requiring treatment, n = 1 surgical site infections, n = 179 non-detailed general complications, n = 57 non-detailed minor surgical complications, n = 34 non-detailed major complications requiring intervention

Complications were further categorised according to the GASTRODATA classification [27], with general complications representing 42.4% and surgical complications representing 35.9% of all adverse events. Both types co-occurred in 21.5% of patients, with missing data in 0.2% of cases. Mortality was up to threefold higher among patients experiencing both general and surgical complications. Non-surgical infections (4.7%; n = 73), anastomotic leak (4.6%; n = 70), abnormal fluid drainage (3.4%; n = 52), and postoperative bleeding requiring invasive treatment (2.6%; n = 40) were the most frequent causes of morbidity. A detailed description of 90-day morbidity and mortality is provided in Table 5.

### Univariate analysis for 90-day morbidity and mortality

On univariate analysis, several pre-operative factors were significantly associated with increased 90-day clinically relevant morbidity and mortality (Clavien–Dindo grade II–V), including older age, higher Charlson Comorbidity Index (CCI), ASA score of 3 or 4, ECOG performance status of 3–4, and severe pre-operative weight loss. ECOG 3–4 was associated with clinically relevant morbidity and mortality outcomes, presenting twice the risk of CD II-V outcome. Surgical variables such as longer operative duration, positive resection margins (R1/R2), and pathologically confirmed metastatic disease (pM1) were also associated with worse outcomes. BMI, gender, and surgical approach (open vs minimally invasive) were not associated with 90-day clinically relevant morbidity and mortality.

### Multivariate analysis for 90-day morbidity and mortality

On multivariate analysis, 90-day clinically relevant morbidity and mortality was significantly associated with pre-operative higher CCI [(*p* < 0.001), (OR = 1.12), (95% CI = 1.05–1.18)], ASA score 3 or 4 [(*p* = 0.001), (OR = 1.55), (95% CI = 1.19–2.02)], severe pre-operative weight loss [(*p* = 0.042), (OR = 1.29), (95% CI = 1.0–1.6)] and surgical determinants such as pathological staging with confirmed metastatic disease [(*p* = 0.038), (OR = 1.79), (95% CI = 1.03–3.10)] and positive (R1 or R2) margin [(*p* = 0.018), (OR = 1.59), (95% CI = 1.08–2.32)]. Age, ECOG performance status, procedure duration, total number of harvested lymph nodes and surgical approach (open vs minimally invasive) were not associated with 90-day clinically relevant morbidity and mortality on multivariate analysis. Details of the analysis are presented in Table 6.

**Table 6:** Univariate and multivariate logistic regression analysis for predicting clinically relevant morbidity and mortality (Clavien Dindo II-V) (n = 1538)

PREDICTORs OF 90-DAY CLINICALLY RELEVANT MORBIDITY AND MORTALITY	Univariate analysis		Multivariate analysis	
	Odds Ratio	*P* value (< 0.05)	Odds Ratio	*P* value (< 0.05)
Age	1.013 (1.005–1.022)	< 0.001	1.004 (0.993–1.016)	0.46
Gender				
Female	1.00 (reference)	0.27		
Male	1.137 (0.904–1.431)			
ECOG Performance				
Status 0–2	1.00 (reference)	< 0.001	-	0.23
Status 3–4	2.322 (1.534–3.516)		1.347 (0.827–2.192)	
Body Mass Index (BMI)	0.999 (0.976–1.023)	0.95	-	
Charlson Comorbidity Index (CCI)	1.200 (1.142–1.261)	< 0.001	1.117 (1.055 – 1.184)	**< 0.001**
ASA Score				
1 or 2	1.00 (reference)	< 0.001	-	**< 0.001**
3 or 4	2.017 (1.612–2.523)		1.554 (1.193 – 2.026)	
Preoperative > 10% Weight Loss				
No	1.00 (reference)	< 0.001	-	**0.04**
Yes	1.487 (1.190–1.857)		1.287 (1.010 – 1.641)	
Total number of harvested Lymph nodes	0.993 (0.985–1.000)	0.06	0.990 (0.982 – 0.998)	**0.02**
Duration of Surgery (minutes)	1.003 (1.002–1.004)	< 0.001	1.004 (1.003 – 1.005)	**< 0.001**
Pathology Anatomic Stage (a)				
0, Ia and Remission	1.00 (reference)		-	
Ib-IIIc	1.200 (0.888–1.622)	0.24	1.041 (0.753–1.440)	0.81
IV	2.791 (1.713–4.548)	< 0.001	1.791 (1.034 – 3.103)	**0.04**
Surgical approach				
Open	1.00 (reference)	0.11		
Minimally invasive	0.834 (0.669–1.039)			
Margin Status				
R0	1.00 (reference)	< 0.001	-	**0.02**
R1 or R2	1.823 (1.311–2.534)		1.586 (1.083 – 2.323)	

Values in parentheses are 95 per cent confidence intervals; ECOG Eastern Cooperative Oncology Group

(a) James D. Brierley (Editor) MKG (Editor), CW (Editor). TNM Classification of Malignant Tumours, 8th Edition. Wiley-Blackwell; 2017

Most deaths were associated with general complications (77.8%), and almost half of all 90-day follow-up deaths had both surgical and general complications occurring in the postoperative period. Anastomotic leak (non-duodenal) was the leading cause of death, representing 24.4% (n = 11) of all mortality (n = 45), 1.5 times more than duodenal leaks.

The median hospital stay was 8 days (IQR 6–11). The readmission rate was 10.6%, and the 90-day reoperation rate was 8.1% (n = 124). Reoperations were mainly performed for non-duodenal anastomotic leaks (31.5%), postoperative haemorrhage (16.9%), and duodenal leaks (10.5%).

## Discussion

**Chart 1:**
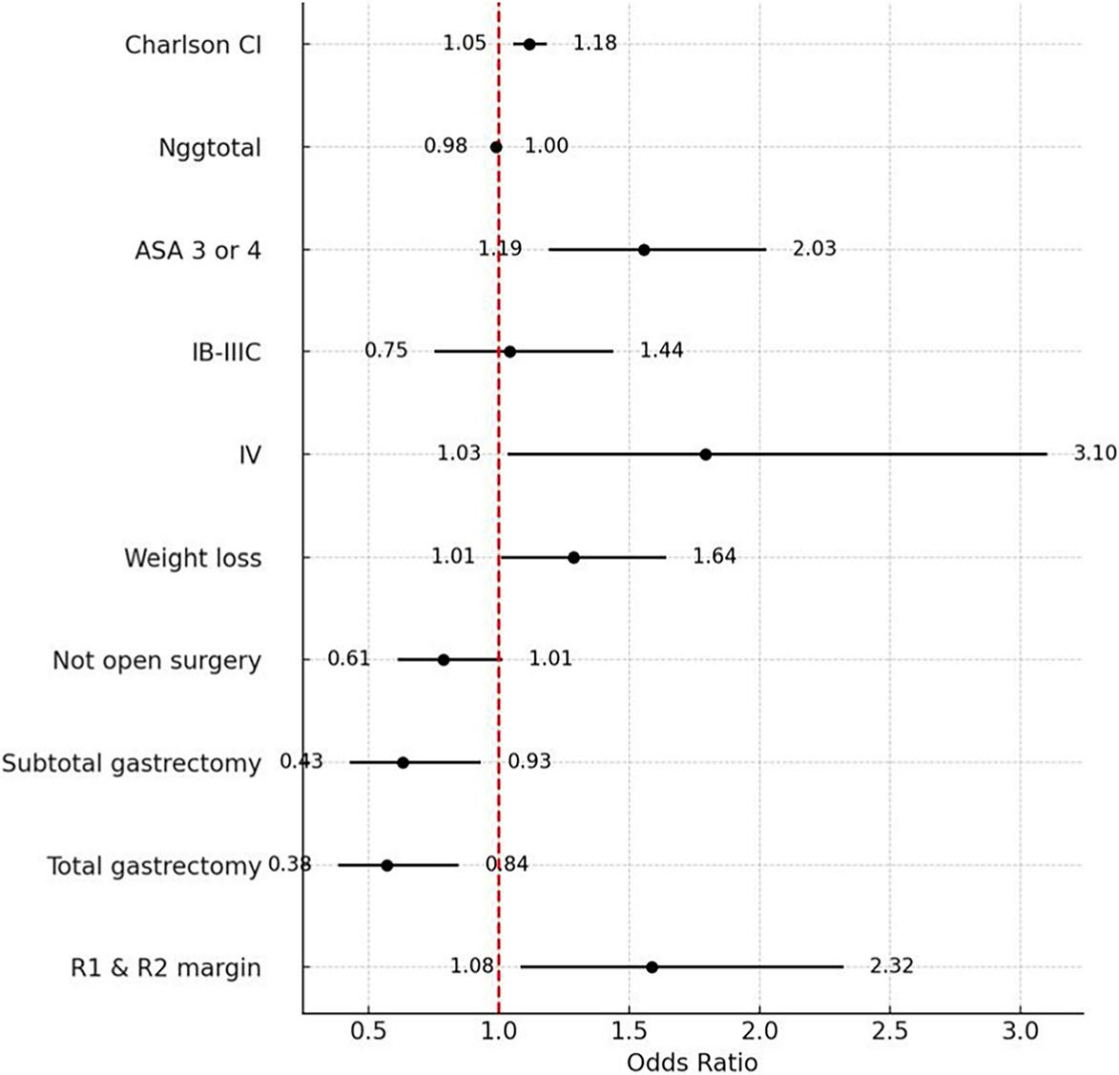
Forest plot representing the odds ratio of multivariate analysis of each specific variable's impact on the outcome (90-day morbidity and mortality, CD II-V). Error bars indicate the confidence intervals (CI). *Ngg Total* Total number of harvested Lymph Nodes. *Ib-IIIc and IV* reporting AJCC TNM Staging

This large, multinational, prospective study of 1538 gastric cancer patients found that elective gastric cancer surgery with curative intent has a 90-day morbidity and mortality of 38.2% (n = 587) and 2.9% (n = 45), respectively. We found that higher CCI, ASA score 3 or 4, severe pre-operative weight loss pathologically confirmed metastatic disease (stage IV), and positive (R1 or R2) margins are significantly associated with clinically relevant 90-day morbidity and mortality, as showed in Chart 1. Additionally, a higher number of lymph nodes retrieved emerged as a potential protective factor in the final model; however, the effect was marginal and not clinically meaningful, as the number of harvested lymph nodes was only weakly associated with reduced odds of 90-day clinically relevant morbidity and mortality (OR = 0.99; 95% CI = 0.982–0.998). Likewise, procedure duration, with an odds ratio close to 1, presented no meaningful association with 90-day morbidity and mortality.

The 90-day morbidity rate of 38.2% was consistent with previous studies, ranging from 6.3% to 43% [13, 19, 20]. Cats et al*.* reported lower 30-day morbidity rates at 22–28% [39], and Baiocchi et al. reported a 90-day morbidity rate of 29.8% [40]. However, our study reported lower clinically significant morbidity rates (CD II-IV) (26.2%) compared to these studies, including the PRESTO study [26], with fewer events per patient (1.3 vs. 1.5) [40]. Non-surgical infections (4.7%) and anastomotic leak (n = 70) were the most common causes of morbidity, as observed in other studies [13, 32].

Our study reported a 2.9% mortality rate, which is lower than previously reported figures, including 3.4% in Peltrini et al. [41] and a range of up to 12.5% in Baiocchi et al. [40]. This may reflect advances in perioperative care and surgical techniques, as observed in more recent prospective [23, 28, 42, 43] and randomised studies [21], suggesting an overall trend toward improved contemporary outcomes.

The reoperation rate was 8.1% in our study, similar to previous studies [40]. The higher comorbidity burden in our cohort may have contributed to a higher 90-day morbidity and mortality rate (Table 6). Although our cohort had a high comorbidity burden, as measured by the Charlson Comorbidity Index (CCI), the 90-day mortality rate was lower than that reported in previous studies [28, 29, 44], suggesting that high comorbidity did not necessarily translate into higher mortality in our population. This finding is consistent with reports highlighting the role of pre-operative prehabilitation and careful patient selection [7, 15]. Such strategies are increasingly recognised for their role in optimising care for high-risk patients and potentially mitigating the impact of comorbidities on surgical outcomes.

Advanced disease defined as metastatic involvement identified intraoperatively or on final pathology (p = 0.0001), and positive surgical margin (p = 0.0004) were both significantly associated with clinically relevant 90-day morbidity and mortality, with almost threefold increase in risk. These findings highlight the importance of carefully balancing the extent of surgical intervention against palliative approaches, taking into account optimal pre-operative staging, patient life expectancy, and individual expectations. In this cohort, patients were deemed suitable for surgery based on ASA and ECOG criteria. However, over half of the cohort had a CCI of 5 or higher. Recognised surgical quality indicators [13, 32] such as R0 resection, D2 lymphadenectomy, and retrieval of > 15 lymph nodes were achieved in the present cohort in 89.1%, 63.1% and 79.1% of the patients, respectively.

In the Western world, unlike in the East, the absence of screening protocols results in the rare early detection of gastric cancer [44]. In our study, locally advanced gastric cancers (stage Ib and above) accounted for the majority of patients (74.2%; n = 1141) at postoperative pathological assessment.

The approach to intra-abdominal complications primarily involved conservative management, similar to previous studies [29], with 21.9% (n = 80) of cases undergoing drainage via endoscopic or interventional radiology. A smaller proportion (8.1%) required reoperation during the same hospital admission. These patients had a significantly higher mortality rate (*p* < 0.001), likely reflecting the severity of the underlying complication and the additional risk associated with re-exploration in clinically unstable individuals.

This study also highlighted differences in treatment and outcomes between duodenal and non-duodenal leaks [40], with the latter necessitating more frequent (55.7%) reoperations and more extended hospital stays (with an average delay of 9 days). Non-duodenal leakage has been reported in previous studies, with a reported incidence ranging from 2 to 11%. It was the leading cause of death in our study, representing 24.4% of all mortality, 1.5 times more than duodenal leaks.

Clavien–Dindo grade ≥ II complications were the primary determinant of prolonged hospital stay (mean > 14 days), particularly in patients undergoing total gastrectomy or experiencing an anastomotic leak (mean stay of 28.1 ± 19.7 days). The overall readmission rate was low (10.6%) and was most related to intra-abdominal collections following subtotal gastrectomy.

Although age was significantly associated with clinically relevant 90-day morbidity and mortality in univariate analysis, as seen in previous studies in the literature [45, 46], it did not remain significant in the multivariate model (p = 0.078). This may reflect confounding or collinearity with other age-related variables such as ASA score and Charlson Comorbidity Index, which remained independently associated with adverse outcomes and may better capture physiological vulnerability in this cohort [47, 48].

Notably, patients aged ≥ 70 years had nearly double the 90-day mortality rate (5.1%) compared to their younger counterparts, reinforcing the clinical relevance of age despite statistical adjustment. Among patients who were ≥ 70 years old and died, 96.8% (n = 18) had a CCI ≥ 5, and most also presented with severe preoperative weight loss.

Ensuring high standards of surgical care involves not only reducing complication rates but also effectively managing complications when they occur, thereby preventing their escalation to critical events or death. The observed combination of significant morbidity and low mortality in our cohort reflects this capacity to 'rescue' patients — a recognised quality indicator in modern surgical practice [7].

## Strengths and weaknesses

This study offers a comprehensive and contemporary overview of real-world 90-day morbidity and mortality after curative gastric cancer surgery, using prospective data from a broad and diverse international cohort. Its novelty lies in the combination of scale, geographical diversity, and standardised complication classification, enabling the identification of clinically relevant predictors across different surgical approaches and health systems. Rather than focusing on a single technique or setting, it reflects current global practice and provides meaningful insights into perioperative risk factors applicable across varied contexts. However, data inconsistencies may arise from structural database flaws or institutional apprehension about high complication rates and inadequate follow-up. The classification of lymphadenectomy as D1, D1 + or D2 was based on the operating surgeon’s intraoperative judgement and documentation, without central validation. This subjective assessment may not always correspond to the final number of lymph nodes retrieved, which is also influenced by pathological processing and institutional practices. Variability in patient demographics contributed to wide standard deviations, and the absence of systematic data on patient optimisation before reintervention and on the timing of such interventions limits our ability to assess their impact on outcomes. Given the heterogeneity across participating institutions and the absence of standardised treatment pathways, our findings should be interpreted as a reflection of real-world practice. Future research should aim to examine how institutional characteristics and care models impact surgical outcomes in gastric cancer. Comparative studies between Eastern and Western treatment strategies would be valuable.

## Conclusion

Elective gastric cancer surgery was associated with 90-day morbidity of 38.2%, with a total of 26.2% classified as clinically relevant (CD II-IV), and 90-day mortality of 2.9% in this global cohort. Factors associated with this morbidity and mortality (CD II-V) included a higher CCI, a higher ASA score, pre-operative severe weight loss, pathological confirmation of metastatic disease, and a positive margin. These findings reflect real-world variability and underscore the importance of understanding how institutional and regional factors influence surgical outcomes.

## Supplementary Information

Below is the link to the electronic supplementary material.

Supplementary file1 (PDF 446 KB)

## Data Availability

Data is available upon request from corresponding author.
