# Supplementary material for: Stomach cancer elective surgery morbidity and mortality at 90-Day (Hold Study): a prospective, international collaborative cohort study
Source: Langenbecks Arch Surg. 2025 Nov 14;411(1):12. doi: 10.1007/s00423-025-03890-7 (PMC12618408; doi:10.1007/s00423-025-03890-7)
Supplement: Supplementary file 1 — Supplementary file1 (PDF 446 KB) [file 423_2025_3890_MOESM1_ESM.pdf]

HOLD Study | TMNS

Data Dictionary

Stomac**H** Cancer Elective Surgery M**O**rbidity and Morta**L**ity at 90-**D**ay

ID/DEMOGRAPHY

EXCEL COLUMN	QUESTION	ANSWER OPTIONS	DICTIONARY | Where to find DATA
A	Country Name	Open Text	Country where your patient institution is located – please don’t name the city and avoid abbreviations
B	Institution Name	Open Text	Please use your Official Institution Name as available in the Internet Site or national reference – Please avoid abbreviations
C	Patient Serial Number	Open Text	This is the number of each sequential patients included in the HOLD study in one institution
D	Patient Unit/ Hospital Identification Number	Open Text	The official Hospital/institution number of each patient just for Collaborator during patient follow up and data collection – DON’T share it, DON’T submit this field
E	Ethnicity Code of the patient	Please choose one: 1= Caucasian, 2 = Afro-Caribbean, 3 = Asian, 4 = Hispanic, 5 = Other	-
F	Sex	Please choose one: 1=Male, 2= Female, 3 = Other	-
G	Age at the time of surgery	Please insert patient age in years	DON’T insert patient birth date

Clinical DATA

EXCEL COLUMN	QUESTION	ANSWER OPTIONS	DICTIONARY | WHERE TO FIND DATA
H	At surgery/Most recent BMI	Insert value - Units: Kg/m2	Please insert the most recent Body Mass index (BMI) that you have access, ideally calculated in the day of the surgery.<br>**Where:** Usually present in Nurses Information at admission, previous Outpatient info or Anaesthesiologists' Data
I	American Society of Anesthesiologists (ASA) Score	Please choose the ASA Score: 1= ASA1; 2=ASA 2; 3=ASA 3; 4= ASA 4	**Where:** Usually available in Anaesthesiologists' Data
J	Charlson Comorbidity Index	Insert Value - Calculator: https://www.mdcalc.com/charlson-comorbidity-index-cci	**Where:** Usually available in Anaesthesiologists' Data.<br>If <50 years old and no previous or present diseases the patient is a **+2** (Solid Organ)
K	Weight loss >10% last 6 months?	Please choose one: 0=No; 1=Yes	**Where:** Usually present in Nurses Information at admission, previous Outpatient info or Anaesthesiologists' Data
L	Performance Status from Eastern Cooperative Oncology Group (ECOG)	Calculator: https://www.mdcalc.com/eastern-cooperative-oncology-group-ecog-performance-status<br><br>Please choose one:<br>0 = PS0 (0 points)<br>1= PS1 (1 points)<br>2= PS2 (2 points)<br>3= PS3 (3 points)<br>4= PS4 (4 points)	Grade Performance Status ECOG<br>0- Fully active, able to carry on all pre-disease performance without restriction<br>1- Restricted in physically strenuous activity but ambulatory and able to carry out work light or sedentary<br>2- Ambulatory and capable of all selfcare but unable to carry out any work activities. Up and about more than 50% of waking hours<br>3- Capable of only limited selfcare, confined to bed or chair more than 50% of waking hours<br>4- Completely disabled. Cannot carry on any selfcare. Totally confined to bed or chair<br>*(Reference: Oken MM, Creech RH, Tormey DC, Horton J, Davis TE, McFadden ET, Carbone PP. Toxicity and response criteria of the Eastern Cooperative Oncology Group. Am J Clin Oncol. 1982 Dec;5(6):649-55. PMID: 7165009)*<br>**Where:** Usually present in Nurses Information at admission, previous Outpatient info or Anaesthesiologists' Data

Oncological DATA

EXCEL COLUMN	QUESTION	ANSWER OPTIONS	DICTIONARY | WHERE TO FIND DATA
M	**Pre-operative Histology known?**	Please Choose:<br>0=No;<br>1=Yes	Confirm if there was a prior to surgery biopsy of the gastric tumour (i.e., by endoscopy) with a conclusive diagnosis<br>**Where:** Usually present in Nurses Information at admission, previous Outpatient info or Oncologist Data
N/O/P	**cTNM 8th Edition (*Pre-Surgery* Staging)**	cT (0=T0; 1=T1; 2=T2; 3=T3; 4=T4; 5=Tis; 6=Tx)<br>cN (0=N0;1=N1; 2=N2; 3=N3; 4=Nx)<br>cM (0=M0; 1=M1;2=Mx)	*https://www.uicc.org/resources/tnm-classification-malignant-tumours-8th-edition*<br><br>For descriptive we suggest: *https://www.cancer.org/cancer/stomach-cancer/detection-diagnosis-staging/staging.html*
Q	**Neoadjuvant Chemotherapy?**	Please Choose:<br>0=No;<br>1=Yes	0=No → not proposed or not even began before surgery<br>1=Given, but not completed → the patient started therapy but it was stop before completing all cycles<br>2= Given and completed → all cycles of therapy were completed and<br>**Where:** Usually present in Nurses Information at admission, previous Outpatient info or Oncologist Data
R	**Neoadjuvant Radiotherapy?**	Please Choose:<br>0=No;<br>1=Yes	0=No → not proposed or not even began before surgery<br>1=Given, but not completed → the patient started therapy but it was stop before completing all cycles<br>2= Given and completed → all cycles of therapy were completed<br>**Where:** Usually present in Nurses Information at admission, previous Outpatient info or Oncologist Data

Surgical DATA

Surgical Procedure

EXCEL COLUMN	QUESTION	ANSWER OPTIONS	DICTIONARY | Where to find DATA
S	**Surgical Approach**	Please choose one:<br>0=open<br>1=Laparoscopic<br>2=Laparoscopic converted to open<br>3= Robotic;<br>4= Robotic converted to open	0=open → Laparotomy<br>1=Laparoscopic<br>2=Laparoscopic converted to open→ surgery starting by laparoscopy with need to conversion to open surgery<br>3= Robotic;<br>4= Robotic converted to open→ surgery starting by robotic with need to conversion to open surgery<br>**Where:** Operating Room registrations
T	**Type of Resection** (Type of stomach resection)	Please choose one:<br>0=Proximal gastrectomy<br>1= Subtotal gastrectomy<br>2=Total Gastrectomy<br>3=Extended Total Gastrectomy<br>4=Esophago-gastrectomy<br>5=Wedge Resection	0=Proximal gastrectomy → Stomach resection including the cardia (esophagogastric junction). The pylorus is preserved.<br>1= Subtotal gastrectomy → Stomach resection including the pylorus. The cardia is preserved. In the standard gastrectomy, two-third of the stomach is resected.<br>2=Total Gastrectomy → Total resection of the stomach including the cardia and pylorus.<br>3=Extended Total Gastrectomy → A non-standard gastrectomy in which (1) Gastrectomy with combined resection of adjacent involved organs OR (2) Gastrectomy with extended lymphadenectomy exceeding D2.<br>4=Esophago-gastrectomy → Total gastrectomy with lower oesophageal resection<br>5=Wedge Resection → non-circumferential resection of a segment of the stomach<br>(*Reference: Japanese gastric cancer treatment guidelines 2018 (5th edition)* *https://doi.org/10.1007/s10120-020-01042-y*)<br><br>**Where:** Operating Room registrations
U	**Reconstruction Type**	Please Choose one:<br>0=Roux em Y;<br>1=Bilroth I;<br>2=Bilroth II;<br>3= Jejunal interposition;<br>4= Colonic interposition;<br>5=Gastric reconstruction<br>6=No reconstruction	How the anastomosis has been done<br>0=Roux em Y;<br>1=Bilroth I;<br>2=Bilroth II;<br>3= Jejunal interposition;<br>4= Colonic interposition;<br>5=Gastric reconstruction → includes i.e., stapling after wedge resection<br>6=No reconstruction → no anastomosis after resection i.e., complication during surgery with need for abrupt stop and 2nd look<br>**Where:** Operating Room registrations

V	**Lymphadenectomy**	Please choose one:<br>0=D0<br>1=D1<br>2=D2<br>3=D1+<br>4=D2+	0=D0 → Lymphadenectomy less than D1.<br>1=D1 → Stations No. 1–7<br>2=D2 → Stations D1 + No. 8a, 9, 11p, 11d, 12a AND includes Nos. 19, 20, 110* and 111For tumours invading the oesophagus<br>3=D1+ → Stations D1 + No. 8a, 9, 11p AND includes No.<br>110* For tumours invading the oesophagus<br>4=D2+ → Stations beyond D2<br>*(Reference: Japanese gastric cancer treatment guidelines 2018 (5th edition) - https://doi.org/10.1007/s10120-020-01042-y)*<br>**Where:** Operating Room registrations
W	**Feeding jejunostomy**	Please Choose one:<br>0=No<br>1=Yes	If the chosen option was a naso-gastric or naso-jejunal tube (for feeding or drainage) please select 0=No<br>**Where:** Operating Room registrations
X	**HIPEC**	Please Choose one:<br>0=No<br>1=Yes	Hyperthermic intraperitoneal chemotherapy (HIPEC)<br>In case of PIPAC the patient should be EXCLUDED from the present study<br>**Where:** Operating Room registrations
Y	**Procedure duration - "skin to skin time"**	Insert value (Time in minutes)	Complete time of the procedure, since first incision until completion of wound(s) closure, in minutes<br>**Where:** Operating Room registrations
Z	**Intraoperative complication?**	Please Choose one:<br>0=No<br>1=Yes	During Surgery was there any "any deviation from the ideal intraoperative course occurring between skin incision and skin closure". Any event during the surgery should be considered "regardless of whether it is related to surgery or anaesthesia and regardless of pre-existing risk factors (...)".<br>Events NOT defined as intraoperative complications:<br>1. Sequelae, i.e., effects inherent to current best practice surgery<br>2. Failures of cure (such as residual tumour after surgery)<br>3. Events related to the underlying disease (encasement of splenic artery with need for splenectomy during index procedure)<br>4. Wrong-site or wrong-patient surgery or errors in indication<br>5. A deviation from the planned intervention due to unexpected intraoperative findings in conform to current guidelines."<br>"Complications evident only after skin closure are captured with the classification for postoperative complications".<br>*(Reference: World J Surg. 2015 Jul;39(7):1663-71. doi: 10.1007/s00268-015-3003-y)*<br>**Where:** Operating Room registrations
AA	**If Yes, please name of Intraoperative Complication**	Insert intraoperative complication	Use a single word if possible. Avoid abbreviations and try to use common surgical/medical nomenclature.

Pathology Report

EXCEL COLUMN	QUESTION	ANSWER OPTIONS	DICTIONARY | WHERE TO FIND DATA
AB	**Tumour Histology (WHO)**	Please Choose one:<br>0=Adenocarcinoma<br>1=Adenosquamous<br>2=Carcinoma with lymphoid stroma<br>3=Hepatoid carcinoma<br>4=Squamous cell Carcinoma<br>5=Undifferentiated<br>6= GIST<br>7=Other	*(Reference: Cancer Staging UICC & AJCC, 8th Edition)*<br>**Where:** Histology report
AC/AD/AE	**(y)pTNM 8th Edition (Histology *after* resection)[1]**	pT (0=T0; 1=T1; 2=T2; 3=T3; 4=T4; 5=Tis)<br>pN (0=N0;1=N1; 2=N2; 3=N3; 4=Nx)<br>pM (0=M0; 1=M1;2=Mx)	*(Reference: https://www.uicc.org/resources/tnm-classification-malignant-tumours-8th-edition)*
AF	**Resection Margin**	Please choose one<br>0=R0<br>1=R1<br>2=R2	R0 - Complete resection with cancer cell negative margin<br>R1 - Microscopical positive margin with cancer cells presented at the resection margin by pathological examination<br>R2 - Tumour tissue seen at the resection margin on gross examination by the naked eye<br>*(Reference: Jiang Z, et al. BMJ Open 2020;10:e040282. doi:10.1136/bmjopen-2020-040282)*
AG/AH	**Resected Lymph nodes**	Total number	Total number of lymph nodes counted in the Histology evaluation of the resected tissue<br>**Where:** Histology report
		Number of positive lymph nodes	Lymph nodes with metastatic disease<br>**Where:** Histology report

[1] (y) post-therapy Histology classification, measures response to neoadjuvant treatment.

90-DAY POST SURGERY DATA

EXCEL COLUMN	QUESTION	ANSWER OPTIONS	DICTIONARY | WHERE TO FIND DATA
AI	Did the patient DIE within the 90-day of surgery irrespective of the cause of death (all-cause mortality)?	Please Choose one:<br>0=No<br>1=Yes	All-cause mortality – 1=Yes if the patient has died in any day after surgery, from 1st until 90th day, from any cause.
AJ	Did the patient suffer from any COMPLICATION within the 90-day of surgery?	Please Choose one:<br>0=No<br>1=Yes	After Surgery complication: “Complications evident only after skin closure are captured with the classification for postoperative complications”.<br>*(Reference: World J Surg. 2015 Jul;39(7):1663-71. doi: 10.1007/s00268-015-3003-y)*
AK	Did the patient develop any Postoperative GENERAL complications within the 90-day of surgery?	Please Choose one:<br>0=No<br>1=Yes	Complications of not direct surgical cause, such as non-surgical site infections and major respiratory problems - Check *PostopGeneral* (Table 1) to confirm if it is contemplated (last page).
AL	If yes, what was the MAIN Postoperative GENERAL complication?	Please choose one of the complications listed	Check *PostopGeneral* (Table 1) for proper assignment (last page).
AM	Did the patient develop any Postoperative SURGICAL complications within the 90-day of surgery?	Please Choose one:<br>0=No<br>1=Yes	Complications of surgical cause, such as anastomotic leak - Check *PostopSurgical* (Table 2) for proper assignment (last page)
AN	If yes, what was the MAIN Postoperative SURGICAL complication?	Please choose one of the complications listed	Check *PostopSurgical* (Table 2) for proper assignment (last page)
AO	What was the Clavien-Dindo Classification of the complication?	Please choose one grade listed below	Please check *Clavien-Dindo Grade* (Table 3) for proper assignment of worst complication (last page) or use this Calculator: http://shorturl.at/nzCEK
AP	90-day Reoperation?	Please Choose one:<br>0=No<br>1=Yes	Was the patient submitted to a new surgery in the 90 days post index surgery?
AQ	90 day endoscopic/radiological treatment?	Please Choose one:<br>0=No<br>1=Yes	Complication of index procedure with need for endoscopic (i.e., endoluminal bleeding or other) or radiological (i.e., selective embolization or other) in the 90 days post index surgery?

EXCEL COLUMN	QUESTION	ANSWER OPTIONS	DICTIONARY | WHERE TO FIND DATA
AR	**Did patient suffer from symptomatic SARS-CoV-2 infection (COVID-19) perioperatively? (30 days before or after surgery)**	Please Choose one:<br>0=No<br>1=Yes	Symptomatic SARS-CoV-2 infection (COVID-19) - A patient with positive SARS-CoV-2 (COVID-19) test AND:<br>• Acute respiratory illness (fever and at least one sign/symptom of respiratory disease, e.g., cough, shortness of breath) OR<br>• Additional symptoms of severe COVID-19 disease (shortness of breath, loss of appetite, confusion, persistent pain or pressure in the chest) OR<br>• Less common symptoms such as loss of taste or smell, nasal congestion, conjunctivitis, sore throat, headache, muscle or joint pain, different types of skin rash, nausea or vomiting, diarrhoea, chills or dizziness<br>*(Reference: Coronavirus disease 2019 (COVID-19)Situation Report – 73 = https://bit.ly/37HQsGp)*
AS	**If patient has DIED, was the patient ever discharged home after surgery?<br>Please leave blank if Not Applicable.**	Please Choose one:<br>0=No<br>1=Yes	Was the patient ever well enough after surgery, to be discharged and sent home, even if in the end the patient died in the 90 days after surgery?<br>If the patient was ALIVE at 90 days after surgery please leave blank.
AT	**If patient is ALIVE, is the patient still in hospital at 90 days?**	Please Choose one:<br>0=No<br>1=Yes	Did the patient survive but was never well enough to be discharged home after surgery or was readmitted and is still in the hospital at 90 days after surgery?<br>If the patient DIED please leave blank.
AU	**Was the patient ever discharged home?**	Please Choose one:<br>0=No<br>1=Yes	Was the patient ever well enough to be discharged home?
AV	**If he was DISCHARGED HOME, on which postoperative day was the patient discharged?**	Insert the number of the day	Counting the days after surgery, in which day was the patient discharged home?<br>If never discharged or died during 90 days post op please leave blank
AW	**If the patient was ever discharged home, was she/ he readmitted?**	Please Choose one:<br>0=No<br>1=Yes	After discharged, did the patient came back to the index hospital or other hospital was readmitted in a ward?

Table 1 - Postop General Complications spreadsheet	Table 2 - Postop Surgical Complications spreadsheet
1. Non-surgical infections	1. Anastomotic leak
2. Pleural effusion requiring drainage	2. Abnormal fluid from drainage, abdominal collections WITHOUT Leak
3. Respiratory failure requiring reintubation	3. Postoperative bleeding requiring invasive treatment
4. Acute renal insufficiency/renal failure requiring Continuous Veno-Venous Hemofiltration (CVVH)/dialysis	4. Other major complications requiring re-intervention or other invasive procedure
5. Need for prolonged intubation (> 24 hours after surgery)	5. Postoperative bowel obstruction
6. Need for tracheostomy	6. Postoperative pancreatic fistula
7. Need for Cardiopulmonary resuscitation (CPR)	7. Duodenal leak
8. Pulmonary embolism	8. Delayed gastric emptying (>10th postoperative day)
9. Pneumothorax requiring treatment	9. Postoperative pancreatitis
10. Myocardial infarction	10. Postoperative bowel perforation or necrosis
11. Acute myocardial failure with acute pulmonary edema	11. Other - May write the other complication witnessed
12. Cardiac dysrhythmia requiring invasive treatment	
13. Stroke causing patient's permanent deficit	
14. Acute liver dysfunction (Child-Pugh >8 for 48 hours)	
15. Other - May write the other complication witnessed	

(Reference: Baiocchi GL, Giacopuzzi S, Reim D, et al. Incidence and grading of complications after gastrectomy for cancer using the GASTRODATA registry a European retrospective observational study. Annals of Surgery. 2020;272(5):807-813. doi:10.1097/SLA.0000000000004341)

Table 3 - Clavien Dindo Classification	
Grade I	Any deviation from the normal post-operative course not requiring surgical, endoscopic or radiological intervention. This includes the need for certain drugs (e.g., antiemetics, antipyretics, analgesics, diuretics and electrolytes), treatment with physiotherapy and wound infections that are opened at the bedside
Grade II	Complications requiring drug treatments other than those allowed for Grade I complications - includes blood transfusion and total parenteral nutrition (TPN)
Grade III	Complications requiring surgical, endoscopic or radiological intervention
	**Grade IIIa -** intervention not under general anaesthetic
	**Grade IIIb -** intervention under general anaesthetic
Grade IV	Life-threatening complications: this includes CNS complications (e.g., brain haemorrhage, ischemic stroke, subarachnoid haemorrhage) which require intensive care, but excludes transient ischemic attacks (TIAs)
	**Grade IVa -** single-organ dysfunction (including dialysis)
	**Grade IVb -** multi-organ dysfunction
Grade V	Death of the patient

SUBMISSION CHECK LIST

Before Submission – Please Confirm:

- Your data is complete - including 90 day follow up of each patient
- You have removed the Patient Unit/ Hospital Identification Number
- If you used PDF print document that all fields have been correctly completed on the online DATA Spread Sheet (Available in HOLD Study Google Drive - https://bit.ly/HOLDStudy_Folder)
